# Supplementary material for: Structural and Functional Neuroimaging of Visual Hallucinations in Lewy Body Disease: A Systematic Literature Review
Source: Brain Sci. 2017 Jul 15;7(7):84. doi: 10.3390/brainsci7070084 (PMC5532597; doi:10.3390/brainsci7070084)
Supplement: Supplementary file 1 [file brainsci-07-00084-s001.docx]

Supplementary tables

**Table S1.** Suitability assessment criteria used to assess structural and functional imaging studies focusing on VH..

| **Questions** | | **Values** |
| --- | --- | --- |
| **1.** | **Were aims related to VH clearly stated?** | no = 0; yes = 1 |
| **2.** | **Were a priori hypotheses on VH clearly stated?** | no = 0; yes = 1 |
| **3.** | **How large was the sample size for each group? ^1^** | < 15 = 0; ≥ 15 = 1 |
| **4.** | **Were demographic and clinical features clearly stated for LBD patients?** | no = 0; yes = 1 |
| **5.** | **Was a group comparison between LBD patients with and without VH performed?** | no = 0; yes = 1 |
| **6.** | **Were LBD patients with and without VH matched for age, global cognitive performance, and disease duration and/or severity?** | no = 0; yes = 1 |
| **7.** | **Was information on pharmacological treatments reported?** | no = 0; yes = 1 |
| **8.** | **Was a multimodal imaging approach used?** | no = 0; yes = 1 |
| **9.** | **Was a whole brain approach used (as opposed to a ROI approach)?** | no = 0; yes = 1 |
| **10.** | **Was correction for multiple comparisons used for voxel-based analyses?** | no = 0; yes = 1 |
| **11.** | **Were covariates of no interest included in the analysis?** | no = 0; yes = 1 |
| **12.** | **Were correlation analyses with VH indices included?** | no = 0; yes = 1 |
| **13.** | **Were correlation analyses with cognitive measures included?** | no = 0; yes = 1 |
| **14.** | **Were limitations of the studies clearly stated?** | no = 0; yes = 1 |

^1^ For studies performing only correlation analyses, a value of 0 was assigned if the number of patients with VH was not clearly reported.

**Table S2.** Suitability assessment of the neuroimaging studies included. The quality assessment focused on structural and functional neuroimaging imaging analyses related to VH only (excluding single cases).

| **Study** | **1** | **2** | **3** | **4** | **5** | **6** | **7** | **8** | **9** | **10** | **11** | **12** | **13** | **14** | Total |
| --- | --- | --- | --- | --- | --- | --- | --- | --- | --- | --- | --- | --- | --- | --- | --- |
| Blanc et al. (2016) [1] | 0 | 0 | 0 | 1 | 1 | 0 | 1 | 0 | 1 | 0 | 1 | 0 | 0 | 1 | **6** |
| Boecker et al. (2007) [2] | 1 | 0 | 0 | 1 | 1 | 0 | 1 | 0 | 1 | 1 | 1 | 0 | 0 | 0 | **7** |
| Delli Pizzi et al. (2016) [3] | 0 | 0 | 1 | 1 | 0 | 0 | 1 | 0 | 0 | 0 | 1 | 1 | 1 | 1 | **7** |
| Delli Pizzi et al. (2014) [4] | 1 | 0 | 0 | 1 | 0 | 0 | 0 | 0 | 1 | 1 | 1 | 1 | 0 | 0 | **6** |
| Delli Pizzi et al. (2014) [5] | 1 | 1 | 0 | 1 | 0 | 0 | 1 | 0 | 0 | 0 | 1 | 1 | 0 | 1 | **7** |
| Erskine et al. (2015) [6] | 1 | 1 | 0 | 1 | 0 | 0 | 1 | 0 | 0 | 0 | 0 | 0 | 0 | 0 | **4** |
| Firbank et al. (2016) [7] | 1 | 1 | 1 | 1 | 0 | 0 | 1 | 0 | 1 | 1 | 1 | 1 | 0 | 1 | **10** |
| Franciotti et al. (2015) [8] | 1 | 1 | 1 | 1 | 1 | 1 | 1 | 1 | 0 | 0 | 1 | 0 | 0 | 1 | **10** |
| Gama et al. (2014) [9] | 1 | 1 | 0 | 1 | 0 | 0 | 1 | 0 | 0 | 1 | 0 | 0 | 0 | 1 | **6** |
| Gasca-Salas et al. (2016) [10] | 1 | 0 | 0 | 1 | 1 | 1 | 1 | 0 | 1 | 1 | 1 | 0 | 0 | 1 | **9** |
| Goldman et al. (2014) [11] | 1 | 0 | 1 | 1 | 1 | 1 | 1 | 0 | 1 | 0 | 1 | 1 | 0 | 1 | **10** |
| Heitz et al. (2015) [12] | 1 | 1 | 1 | 1 | 1 | 0 | 1 | 0 | 1 | 0 | 1 | 1 | 0 | 1 | **10** |
| Holroyd and Wooten (2006) [13] | 1 | 0 | 0 | 1 | 1 | 0 | 0 | 0 | 1 | 0 | 0 | 0 | 0 | 1 | **5** |
| Ibarretxe-Bilbao et al. (2010) [14] | 1 | 1 | 0 | 1 | 0 | 0 | 1 | 0 | 1 | 1 | 1 | 0 | 1 | 1 | **9** |
| Ibarretxe-Bilbao et al. (2008) [15] | 1 | 1 | 1 | 1 | 1 | 1 | 0 | 0 | 0 | 1 | 0 | 0 | 1 | 1 | **9** |
| Iizuka and Kameyama (2016) [16] | 0 | 0 | 0 | 1 | 0 | 0 | 0 | 1 | 0 | 0 | 0 | 1 | 0 | 1 | **4** |
| Imamura et al. (1999) [17] | 1 | 0 | 0 | 1 | 1 | 0 | 0 | 0 | 0 | 0 | 1 | 0 | 0 | 0 | **4** |
| Janzen et al. (2012) [18] | 1 | 1 | 0 | 1 | 1 | 0 | 1 | 0 | 1 | 1 | 1 | 0 | 0 | 1 | **9** |
| Kantarci et al. (2010) [19] | 0 | 0 | 0 | 1 | 1 | 0 | 0 | 1 | 0 | 0 | 0 | 0 | 0 | 1 | **4** |
| Kantarci et al. (2012) [20] | 0 | 0 | 1 | 1 | 0 | 0 | 0 | 1 | 0 | 0 | 0 | 1 | 0 | 0 | **4** |
| Lee et al. (2016) [21] | 1 | 1 | 0 | 1 | 1 | 1 | 1 | 1 | 0 | 1 | 0 | 0 | 0 | 1 | **9** |
| Lee et al. (2017) [22] | 1 | 0 | 0 | 1 | 1 | 0 | 1 | 1 | 1 | 1 | 1 | 0 | 0 | 1 | **9** |
| Lefebvre et al. (2016) [23] | 1 | 1 | 1 | 1 | 1 | 1 | 1 | 0 | 1 | 1 | 1 | 0 | 0 | 1 | **11** |
| Lobotesis et al. (2001) [24] | 0 | 0 | 0 | 1 | 1 | 0 | 0 | 0 | 0 | 0 | 0 | 0 | 0 | 1 | **3** |
| Matsui et al. (2006) [25] | 1 | 1 | 1 | 1 | 1 | 0 | 1 | 0 | 1 | 0 | 1 | 0 | 0 | 1 | **9** |
| Meppelink et al. (2009) [26] | 1 | 1 | 0 | 1 | 1 | 0 | 1 | 0 | 1 | 1 | 1 | 0 | 0 | 0 | **8** |
| Meppelink et al. (2011) [27] | 1 | 0 | 0 | 0 | 1 | 0 | 0 | 1 | 1 | 1 | 1 | 0 | 0 | 0 | **6** |
| Miyazawa et al. (2010) [28] | 0 | 0 | 0 | 0 | 0 | 0 | 1 | 0 | 0 | 0 | 0 | 1 | 0 | 1 | **3** |
| Nagahama et al. (2010) [29] | 1 | 0 | 1 | 1 | 0 | 0 | 1 | 0 | 0 | 0 | 1 | 1 | 0 | 1 | **7** |

**Table S2 (continued).** Suitability assessment of the neuroimaging studies included. Quality assessment focused on structural and functional neuroimaging imaging analyses related to VH only (excluding single cases).

| **Study** | **1** | **2** | **3** | **4** | **5** | **6** | **7** | **8** | **9** | **10** | **11** | **12** | **13** | **14** | Total |
| --- | --- | --- | --- | --- | --- | --- | --- | --- | --- | --- | --- | --- | --- | --- | --- |
| Nagano-Saito et al. (2004) [30] | 1 | 0 | 0 | 1 | 1 | 1 | 1 | 0 | 0 | 1 | 0 | 0 | 0 | 0 | **6** |
| O'Brien et al. (2005) [31] | 1 | 1 | 0 | 1 | 0 | 0 | 1 | 0 | 1 | 1 | 0 | 1 | 0 | 1 | **8** |
| Oishi et al. (2005) [32] | 1 | 0 | 1 | 1 | 1 | 1 | 1 | 0 | 1 | 1 | 1 | 0 | 0 | 0 | **9** |
| Osaki et al. (2005) [33] | 1 | 0 | 0 | 1 | 1 | 0 | 1 | 0 | 0 | 0 | 0 | 0 | 0 | 1 | **5** |
| Pagonabarraga  et al. (2014) [34] | 1 | 0 | 1 | 1 | 1 | 1 | 1 | 0 | 1 | 0 | 1 | 0 | 0 | 1 | **9** |
| Park et al. (2013) [35] | 1 | 1 | 0 | 1 | 1 | 1 | 1 | 0 | 1 | 0 | 0 | 1 | 0 | 1 | **9** |
| Pasquier et al. (2002) [36] | 0 | 0 | 0 | 1 | 1 | 0 | 0 | 0 | 0 | 0 | 0 | 0 | 0 | 1 | **3** |
| Peraza et al. (2014) [37] | 0 | 0 | 0 | 1 | 0 | 0 | 1 | 0 | 1 | 0 | 1 | 1 | 0 | 1 | **6** |
| Peraza et al. (2015) [38] | 1 | 0 | 0 | 1 | 0 | 0 | 1 | 0 | 1 | 0 | 0 | 1 | 0 | 0 | **5** |
| Pereira et al. (2013) [39] | 1 | 1 | 0 | 1 | 1 | 0 | 1 | 0 | 0 | 0 | 0 | 0 | 1 | 1 | **7** |
| Perneczky et al. (2008) [40] | 1 | 1 | 0 | 1 | 1 | 1 | 1 | 0 | 1 | 0 | 1 | 0 | 0 | 1 | **9** |
| Ramirez-Ruiz et al. (2008) [41] | 1 | 0 | 0 | 1 | 1 | 0 | 1 | 0 | 1 | 1 | 1 | 0 | 0 | 1 | **8** |
| Ramirez-Ruiz et al. (2007) [42] | 1 | 1 | 1 | 0 | 1 | 0 | 1 | 0 | 1 | 1 | 1 | 0 | 0 | 0 | **8** |
| Sanchez-Castaneda et al. (2010) [43] | 1 | 1 | 0 | 1 | 1 | 1 | 1 | 0 | 0 | 1 | 1 | 1 | 0 | 1 | **10** |
| Shin et al. (2012) [44] | 1 | 1 | 1 | 1 | 1 | 1 | 1 | 1 | 1 | 0 | 1 | 0 | 0 | 1 | **11** |
| Shine et al. (2015) [45] | 1 | 1 | 0 | 1 | 1 | 1 | 1 | 0 | 0 | 0 | 0 | 0 | 0 | 0 | **6** |
| Shine et al. (2015) [46] | 1 | 1 | 0 | 1 | 0 | 1 | 1 | 0 | 0 | 0 | 0 | 0 | 0 | 0 | **5** |
| Stebbins et al. (2004) [47] | 1 | 0 | 0 | 1 | 1 | 1 | 1 | 0 | 1 | 0 | 1 | 0 | 0 | 1 | **8** |
| Taylor et al. (2012) [48] | 0 | 0 | 0 | 1 | 0 | 0 | 1 | 1 | 0 | 0 | 0 | 1 | 0 | 0 | **4** |
| Uchiyama et al. (2015) [49] | 1 | 1 | 0 | 1 | 0 | 0 | 1 | 0 | 1 | 0 | 1 | 1 | 0 | 1 | **8** |
| Watanabe et al. (2013) [50] | 1 | 0 | 0 | 1 | 1 | 1 | 1 | 0 | 1 | 0 | 1 | 0 | 0 | 1 | **8** |
| Yao et al. (2016) [51] | 1 | 1 | 0 | 1 | 1 | 1 | 1 | 1 | 0 | 1 | 1 | 0 | 0 | 1 | **10** |
| Yao et al. (2015) [52] | 1 | 1 | 0 | 1 | 1 | 1 | 1 | 0 | 1 | 1 | 0 | 0 | 0 | 1 | **9** |
| Yao et al. (2014) [53] | 1 | 1 | 0 | 1 | 1 | 1 | 1 | 1 | 1 | 1 | 1 | 1 | 0 | 1 | **12** |

# References

1. Blanc, F.; Colloby, S.J.; Cretin, B.; de Sousa, P.L.; Demuynck, C.; O'Brien, J.T.; Martin-Hunyadi, C.; McKeith, I.; Philippi, N.; Taylor, J.P. Grey matter atrophy in prodromal stage of dementia with lewy bodies and alzheimer's disease. *Alzheimers Res Ther* **2016**, *8*, 31, 10.1186/s13195-016-0198-6. Available online: <http://dx.doi.org/10.1186/s13195-016-0198-6>.

2. Boecker, H.; Ceballos-Baumann, A.O.; Volk, D.; Conrad, B.; Forstl, H.; Haussermann, P. Metabolic alterations in patients with parkinson disease and visual hallucinations. *Arch Neurol* **2007**, *64*, 984-988, 10.1001/archneur.64.7.984. Available online: <http://dx.doi.org/10.1001/archneur.64.7.984>.

3. Delli Pizzi, S.; Franciotti, R.; Bubbico, G.; Thomas, A.; Onofrj, M.; Bonanni, L. Atrophy of hippocampal subfields and adjacent extrahippocampal structures in dementia with lewy bodies and alzheimer's disease. *Neurobiol Aging* **2016**, *40*, 103-109, 10.1016/j.neurobiolaging.2016.01.010. Available online: <http://dx.doi.org/10.1016/j.neurobiolaging.2016.01.010>.

4. Delli Pizzi, S.; Franciotti, R.; Tartaro, A.; Caulo, M.; Thomas, A.; Onofrj, M.; Bonanni, L. Structural alteration of the dorsal visual network in dlb patients with visual hallucinations: A cortical thickness mri study. *PLoS One* **2014**, *9*, e86624, 10.1371/journal.pone.0086624. Available online: <http://dx.doi.org/10.1371/journal.pone.0086624>.

5. Delli Pizzi, S.; Maruotti, V.; Taylor, J.P.; Franciotti, R.; Caulo, M.; Tartaro, A.; Thomas, A.; Onofrj, M.; Bonanni, L. Relevance of subcortical visual pathways disruption to visual symptoms in dementia with lewy bodies. *Cortex* **2014**, *59*, 12-21, 10.1016/j.cortex.2014.07.003. Available online: <http://dx.doi.org/10.1016/j.cortex.2014.07.003>.

6. Erskine, D.; Taylor, J.P.; Firbank, M.J.; Patterson, L.; Onofrj, M.; O'Brien, J.T.; McKeith, I.G.; Attems, J.; Thomas, A.J.; Morris, C.M.*, et al.* Changes to the lateral geniculate nucleus in alzheimer's disease but not dementia with lewy bodies. *Neuropathol Appl Neurobiol* **2015**, 10.1111/nan.12249. Available online: <http://dx.doi.org/10.1111/nan.12249>.

7. Firbank, M.J.; Lloyd, J.; O'Brien, J.T. The relationship between hallucinations and fdg-pet in dementia with lewy bodies. *Brain Imaging Behav* **2016**, *10*, 636-639, 10.1007/s11682-015-9434-0. Available online: <http://dx.doi.org/10.1007/s11682-015-9434-0>.

8. Franciotti, R.; Delli Pizzi, S.; Perfetti, B.; Tartaro, A.; Bonanni, L.; Thomas, A.; Weis, L.; Biundo, R.; Antonini, A.; Onofrj, M. Default mode network links to visual hallucinations: A comparison between parkinson's disease and multiple system atrophy. *Mov Disord* **2015**, *30*, 1237-1247, 10.1002/mds.26285. Available online: <http://dx.doi.org/10.1002/mds.26285>.

9. Gama, R.L.; Bruin, V.M.; Tavora, D.G.; Duran, F.L.; Bittencourt, L.; Tufik, S. Structural brain abnormalities in patients with parkinson's disease with visual hallucinations: A comparative voxel-based analysis. *Brain Cogn* **2014**, *87*, 97-103, 10.1016/j.bandc.2014.03.011. Available online: <http://dx.doi.org/10.1016/j.bandc.2014.03.011>.

10. Gasca-Salas, C.; Clavero, P.; Garcia-Garcia, D.; Obeso, J.A.; Rodriguez-Oroz, M.C. Significance of visual hallucinations and cerebral hypometabolism in the risk of dementia in parkinson's disease patients with mild cognitive impairment. *Hum Brain Mapp* **2016**, *37*, 968-977, 10.1002/hbm.23080. Available online: <http://dx.doi.org/10.1002/hbm.23080>.

11. Goldman, J.G.; Stebbins, G.T.; Dinh, V.; Bernard, B.; Merkitch, D.; deToledo-Morrell, L.; Goetz, C.G. Visuoperceptive region atrophy independent of cognitive status in patients with parkinson's disease with hallucinations. *Brain* **2014**, *137*, 849-859, 10.1093/brain/awt360. Available online: <http://dx.doi.org/10.1093/brain/awt360>.

12. Heitz, C.; Noblet, V.; Cretin, B.; Philippi, N.; Kremer, L.; Stackfleth, M.; Hubele, F.; Armspach, J.P.; Namer, I.; Blanc, F. Neural correlates of visual hallucinations in dementia with lewy bodies. *Alzheimers Res Ther* **2015**, *7*, 6, 10.1186/s13195-014-0091-0. Available online: <http://dx.doi.org/10.1186/s13195-014-0091-0>.

13. Holroyd, S.; Wooten, G.F. Preliminary fmri evidence of visual system dysfunction in parkinson's disease patients with visual hallucinations. *J Neuropsychiatry Clin Neurosci* **2006**, *18*, 402-404, 10.1176/jnp.2006.18.3.402. Available online: <http://dx.doi.org/10.1176/jnp.2006.18.3.402>.

14. Ibarretxe-Bilbao, N.; Ramirez-Ruiz, B.; Junque, C.; Marti, M.J.; Valldeoriola, F.; Bargallo, N.; Juanes, S.; Tolosa, E. Differential progression of brain atrophy in parkinson's disease with and without visual hallucinations. *J Neurol Neurosurg Psychiatry* **2010**, *81*, 650-657, 10.1136/jnnp.2009.179655. Available online: <http://dx.doi.org/10.1136/jnnp.2009.179655>.

15. Ibarretxe-Bilbao, N.; Ramirez-Ruiz, B.; Tolosa, E.; Marti, M.J.; Valldeoriola, F.; Bargallo, N.; Junque, C. Hippocampal head atrophy predominance in parkinson's disease with hallucinations and with dementia. *J Neurol* **2008**, *255*, 1324-1331, 10.1007/s00415-008-0885-8. Available online: <http://dx.doi.org/10.1007/s00415-008-0885-8>.

16. Iizuka, T.; Kameyama, M. Cingulate island sign on fdg-pet is associated with medial temporal lobe atrophy in dementia with lewy bodies. *Ann Nucl Med* **2016**, *30*, 421-429, 10.1007/s12149-016-1076-9. Available online: <http://dx.doi.org/10.1007/s12149-016-1076-9>.

17. Imamura, T.; Ishii, K.; Hirono, N.; Hashimoto, M.; Tanimukai, S.; Kazuai, H.; Hanihara, T.; Sasaki, M.; Mori, E. Visual hallucinations and regional cerebral metabolism in dementia with lewy bodies (dlb). *Neuroreport* **1999**, *10*, 1903-1907, Available online: <http://dx.doi.org/>.

18. Janzen, J.; van 't Ent, D.; Lemstra, A.W.; Berendse, H.W.; Barkhof, F.; Foncke, E.M. The pedunculopontine nucleus is related to visual hallucinations in parkinson's disease: Preliminary results of a voxel-based morphometry study. *J Neurol* **2012**, *259*, 147-154, 10.1007/s00415-011-6149-z. Available online: <http://dx.doi.org/10.1007/s00415-011-6149-z>.

19. Kantarci, K.; Avula, R.; Senjem, M.L.; Samikoglu, A.R.; Zhang, B.; Weigand, S.D.; Przybelski, S.A.; Edmonson, H.A.; Vemuri, P.; Knopman, D.S.*, et al.* Dementia with lewy bodies and alzheimer disease: Neurodegenerative patterns characterized by dti. *Neurology* **2010**, *74*, 1814-1821, 10.1212/WNL.0b013e3181e0f7cf. Available online: <http://dx.doi.org/10.1212/WNL.0b013e3181e0f7cf>.

20. Kantarci, K.; Lowe, V.J.; Boeve, B.F.; Weigand, S.D.; Senjem, M.L.; Przybelski, S.A.; Dickson, D.W.; Parisi, J.E.; Knopman, D.S.; Smith, G.E.*, et al.* Multimodality imaging characteristics of dementia with lewy bodies. *Neurobiol Aging* **2012**, *33*, 2091-2105, 10.1016/j.neurobiolaging.2011.09.024. Available online: <http://dx.doi.org/10.1016/j.neurobiolaging.2011.09.024>.

21. Lee, J.Y.; Yoon, E.J.; Lee, W.W.; Kim, Y.K.; Jeon, B. Lateral geniculate atrophy in parkinson's with visual hallucination: A trans-synaptic degeneration? *Mov Disord* **2016**, *31*, 547-554, 10.1002/mds.26533. Available online: <http://dx.doi.org/10.1002/mds.26533>.

22. Lee, W.W.; Yoon, E.J.; Lee, J.Y.; Park, S.W.; Kim, Y.K. Visual hallucination and pattern of brain degeneration in parkinson's disease. *Neurodegener Dis* **2017**, *17*, 63-72, 10.1159/000448517. Available online: <http://dx.doi.org/10.1159/000448517>.

23. Lefebvre, S.; Baille, G.; Jardri, R.; Plomhause, L.; Szaffarczyk, S.; Defebvre, L.; Thomas, P.; Delmaire, C.; Pins, D.; Dujardin, K. Hallucinations and conscious access to visual inputs in parkinson's disease. *Sci Rep* **2016**, *6*, 36284, 10.1038/srep36284. Available online: <http://dx.doi.org/10.1038/srep36284>.

24. Lobotesis, K.; Fenwick, J.D.; Phipps, A.; Ryman, A.; Swann, A.; Ballard, C.; McKeith, I.G.; O'Brien, J.T. Occipital hypoperfusion on spect in dementia with lewy bodies but not ad. *Neurology* **2001**, *56*, 643-649, Available online: <http://dx.doi.org/>.

25. Matsui, H.; Nishinaka, K.; Oda, M.; Hara, N.; Komatsu, K.; Kubori, T.; Udaka, F. Hypoperfusion of the visual pathway in parkinsonian patients with visual hallucinations. *Mov Disord* **2006**, *21*, 2140-2144, 10.1002/mds.21140. Available online: <http://dx.doi.org/10.1002/mds.21140>.

26. Meppelink, A.M.; de Jong, B.M.; Renken, R.; Leenders, K.L.; Cornelissen, F.W.; van Laar, T. Impaired visual processing preceding image recognition in parkinson's disease patients with visual hallucinations. *Brain* **2009**, *132*, 2980-2993, 10.1093/brain/awp223. Available online: <http://dx.doi.org/10.1093/brain/awp223>.

27. Meppelink, A.M.; de Jong, B.M.; Teune, L.K.; van Laar, T. Regional cortical grey matter loss in parkinson's disease without dementia is independent from visual hallucinations. *Mov Disord* **2011**, *26*, 142-147, 10.1002/mds.23375. Available online: <http://dx.doi.org/10.1002/mds.23375>.

28. Miyazawa, N.; Shinohara, T.; Nagasaka, T.; Hayashi, M. Hypermetabolism in patients with dementia with lewy bodies. *Clin Nucl Med* **2010**, *35*, 490-493, 10.1097/RLU.0b013e3181e05dbc. Available online: <http://dx.doi.org/10.1097/RLU.0b013e3181e05dbc>.

29. Nagahama, Y.; Okina, T.; Suzuki, N.; Matsuda, M. Neural correlates of psychotic symptoms in dementia with lewy bodies. *Brain* **2010**, *133*, 557-567, 10.1093/brain/awp295. Available online: <http://dx.doi.org/10.1093/brain/awp295>.

30. Nagano-Saito, A.; Washimi, Y.; Arahata, Y.; Iwai, K.; Kawatsu, S.; Ito, K.; Nakamura, A.; Abe, Y.; Yamada, T.; Kato, T.*, et al.* Visual hallucination in parkinson's disease with fdg pet. *Mov Disord* **2004**, *19*, 801-806, 10.1002/mds.20129. Available online: <http://dx.doi.org/10.1002/mds.20129>.

31. O'Brien, J.T.; Firbank, M.J.; Mosimann, U.P.; Burn, D.J.; McKeith, I.G. Change in perfusion, hallucinations and fluctuations in consciousness in dementia with lewy bodies. *Psychiatry Res* **2005**, *139*, 79-88, 10.1016/j.pscychresns.2005.04.002. Available online: <http://dx.doi.org/10.1016/j.pscychresns.2005.04.002>.

32. Oishi, N.; Udaka, F.; Kameyama, M.; Sawamoto, N.; Hashikawa, K.; Fukuyama, H. Regional cerebral blood flow in parkinson disease with nonpsychotic visual hallucinations. *Neurology* **2005**, *65*, 1708-1715, 10.1212/01.wnl.0000187116.13370.e0. Available online: <http://dx.doi.org/10.1212/01.wnl.0000187116.13370.e0>.

33. Osaki, Y.; Morita, Y.; Fukumoto, M.; Akagi, N.; Yoshida, S.; Doi, Y. Three-dimensional stereotactic surface projection spect analysis in parkinson's disease with and without dementia. *Mov Disord* **2005**, *20*, 999-1005, 10.1002/mds.20463. Available online: <http://dx.doi.org/10.1002/mds.20463>.

34. Pagonabarraga, J.; Soriano-Mas, C.; Llebaria, G.; Lopez-Sola, M.; Pujol, J.; Kulisevsky, J. Neural correlates of minor hallucinations in non-demented patients with parkinson's disease. *Parkinsonism Relat Disord* **2014**, *20*, 290-296, 10.1016/j.parkreldis.2013.11.017. Available online: <http://dx.doi.org/10.1016/j.parkreldis.2013.11.017>.

35. Park, H.K.; Kim, J.S.; Im, K.C.; Kim, M.J.; Lee, J.H.; Lee, M.C.; Kim, J.; Chung, S.J. Visual hallucinations and cognitive impairment in parkinson's disease. *Can J Neurol Sci* **2013**, *40*, 657-662, Available online: <http://dx.doi.org/>.

36. Pasquier, J.; Michel, B.F.; Brenot-Rossi, I.; Hassan-Sebbag, N.; Sauvan, R.; Gastaut, J.L. Value of (99m)tc-ecd spet for the diagnosis of dementia with lewy bodies. *Eur J Nucl Med Mol Imaging* **2002**, *29*, 1342-1348, 10.1007/s00259-002-0919-x. Available online: <http://dx.doi.org/10.1007/s00259-002-0919-x>.

37. Peraza, L.R.; Kaiser, M.; Firbank, M.; Graziadio, S.; Bonanni, L.; Onofrj, M.; Colloby, S.J.; Blamire, A.; O'Brien, J.; Taylor, J.P. Fmri resting state networks and their association with cognitive fluctuations in dementia with lewy bodies. *Neuroimage Clin* **2014**, *4*, 558-565, 10.1016/j.nicl.2014.03.013. Available online: <http://dx.doi.org/10.1016/j.nicl.2014.03.013>.

38. Peraza, L.R.; Taylor, J.P.; Kaiser, M. Divergent brain functional network alterations in dementia with lewy bodies and alzheimer's disease. *Neurobiol Aging* **2015**, *36*, 2458-2467, 10.1016/j.neurobiolaging.2015.05.015. Available online: <http://dx.doi.org/10.1016/j.neurobiolaging.2015.05.015>.

39. Pereira, J.B.; Junque, C.; Bartres-Faz, D.; Ramirez-Ruiz, B.; Marti, M.J.; Tolosa, E. Regional vulnerability of hippocampal subfields and memory deficits in parkinson's disease. *Hippocampus* **2013**, *23*, 720-728, 10.1002/hipo.22131. Available online: <http://dx.doi.org/10.1002/hipo.22131>.

40. Perneczky, R.; Drzezga, A.; Boecker, H.; Forstl, H.; Kurz, A.; Haussermann, P. Cerebral metabolic dysfunction in patients with dementia with lewy bodies and visual hallucinations. *Dement Geriatr Cogn Disord* **2008**, *25*, 531-538, 10.1159/000132084. Available online: <http://dx.doi.org/10.1159/000132084>.

41. Ramirez-Ruiz, B.; Marti, M.J.; Tolosa, E.; Falcon, C.; Bargallo, N.; Valldeoriola, F.; Junque, C. Brain response to complex visual stimuli in parkinson's patients with hallucinations: A functional magnetic resonance imaging study. *Mov Disord* **2008**, *23*, 2335-2343, 10.1002/mds.22258. Available online: <http://dx.doi.org/10.1002/mds.22258>.

42. Ramirez-Ruiz, B.; Marti, M.J.; Tolosa, E.; Gimenez, M.; Bargallo, N.; Valldeoriola, F.; Junque, C. Cerebral atrophy in parkinson's disease patients with visual hallucinations. *Eur J Neurol* **2007**, *14*, 750-756, 10.1111/j.1468-1331.2007.01768.x. Available online: <http://dx.doi.org/10.1111/j.1468-1331.2007.01768.x>.

43. Sanchez-Castaneda, C.; Rene, R.; Ramirez-Ruiz, B.; Campdelacreu, J.; Gascon, J.; Falcon, C.; Calopa, M.; Jauma, S.; Juncadella, M.; Junque, C. Frontal and associative visual areas related to visual hallucinations in dementia with lewy bodies and parkinson's disease with dementia. *Mov Disord* **2010**, *25*, 615-622, 10.1002/mds.22873. Available online: <http://dx.doi.org/10.1002/mds.22873>.

44. Shin, S.; Lee, J.E.; Hong, J.Y.; Sunwoo, M.K.; Sohn, Y.H.; Lee, P.H. Neuroanatomical substrates of visual hallucinations in patients with non-demented parkinson's disease. *J Neurol Neurosurg Psychiatry* **2012**, *83*, 1155-1161, 10.1136/jnnp-2012-303391. Available online: <http://dx.doi.org/10.1136/jnnp-2012-303391>.

45. Shine, J.M.; Keogh, R.; O'Callaghan, C.; Muller, A.J.; Lewis, S.J.; Pearson, J. Imagine that: Elevated sensory strength of mental imagery in individuals with parkinson's disease and visual hallucinations. *Proc Biol Sci* **2015**, *282*, 20142047, 10.1098/rspb.2014.2047. Available online: <http://dx.doi.org/10.1098/rspb.2014.2047>.

46. Shine, J.M.; Muller, A.L.; O’Callaghan, C.; Hornberger, M.; Halliday, G.M.; Lewis, S.J.G. Abnormal connectivity between the default mode and the visual system underlies the manifestation of visual hallucinations in parkinson’s disease: A task-based fmri study. *npj Parkinson's Disease* **2015**, doi:10.1038/npjparkd.2015.3. Available online: <http://www.nature.com/articles/npjparkd20153>.

47. Stebbins, G.T.; Goetz, C.G.; Carrillo, M.C.; Bangen, K.J.; Turner, D.A.; Glover, G.H.; Gabrieli, J.D. Altered cortical visual processing in pd with hallucinations: An fmri study. *Neurology* **2004**, *63*, 1409-1416, Available online: <http://dx.doi.org/>.

48. Taylor, J.P.; Firbank, M.J.; He, J.; Barnett, N.; Pearce, S.; Livingstone, A.; Vuong, Q.; McKeith, I.G.; O'Brien, J.T. Visual cortex in dementia with lewy bodies: Magnetic resonance imaging study. *Br J Psychiatry* **2012**, *200*, 491-498, 10.1192/bjp.bp.111.099432. Available online: <http://dx.doi.org/10.1192/bjp.bp.111.099432>.

49. Uchiyama, M.; Nishio, Y.; Yokoi, K.; Hosokai, Y.; Takeda, A.; Mori, E. Pareidolia in parkinson's disease without dementia: A positron emission tomography study. *Parkinsonism Relat Disord* **2015**, *21*, 603-609, 10.1016/j.parkreldis.2015.03.020. Available online: <http://dx.doi.org/10.1016/j.parkreldis.2015.03.020>.

50. Watanabe, H.; Senda, J.; Kato, S.; Ito, M.; Atsuta, N.; Hara, K.; Tsuboi, T.; Katsuno, M.; Nakamura, T.; Hirayama, M.*, et al.* Cortical and subcortical brain atrophy in parkinson's disease with visual hallucination. *Mov Disord* **2013**, *28*, 1732-1736, 10.1002/mds.25641. Available online: <http://dx.doi.org/10.1002/mds.25641>.

51. Yao, N.; Cheung, C.; Pang, S.; Shek-kwan Chang, R.; Lau, K.K.; Suckling, J.; Yu, K.; Ka-Fung Mak, H.; Chua, S.E.; Ho, S.L.*, et al.* Multimodal mri of the hippocampus in parkinson's disease with visual hallucinations. *Brain Struct Funct* **2016**, *221*, 287-300, 10.1007/s00429-014-0907-5. Available online: <http://dx.doi.org/10.1007/s00429-014-0907-5>.

52. Yao, N.; Pang, S.; Cheung, C.; Chang, R.S.; Lau, K.K.; Suckling, J.; Yu, K.; Mak, H.K.; McAlonan, G.; Ho, S.L.*, et al.* Resting activity in visual and corticostriatal pathways in parkinson's disease with hallucinations. *Parkinsonism Relat Disord* **2015**, *21*, 131-137, 10.1016/j.parkreldis.2014.11.020. Available online: <http://dx.doi.org/10.1016/j.parkreldis.2014.11.020>.

53. Yao, N.; Shek-Kwan Chang, R.; Cheung, C.; Pang, S.; Lau, K.K.; Suckling, J.; Rowe, J.B.; Yu, K.; Ka-Fung Mak, H.; Chua, S.E.*, et al.* The default mode network is disrupted in parkinson's disease with visual hallucinations. *Hum Brain Mapp* **2014**, *35*, 5658-5666, 10.1002/hbm.22577. Available online: <http://dx.doi.org/10.1002/hbm.22577>.
